# Hepatoprotective Effect and Chemical Assessment of a Selected Egyptian Chickpea Cultivar

**DOI:** 10.3389/fphar.2016.00344

**Published:** 2016-09-28

**Authors:** Reham H. Mekky, Mostafa R. Fayed, Mohamed R. El-Gindi, Azza R. Abdel-Monem, María del Mar Contreras, Antonio Segura-Carretero, Essam Abdel-Sattar

**Affiliations:** ^1^Department of Pharmacognosy, Faculty of Pharmacy, Egyptian Russian UniversityCairo, Egypt; ^2^Department of Pharmacology and Toxicology, Faculty of Pharmacy, Egyptian Russian UniversityCairo, Egypt; ^3^Department of Pharmacognosy, Faculty of Pharmacy, Cairo UniversityCairo, Egypt; ^4^Department of Analytical Chemistry, Faculty of Sciences, University of GranadaGranada, Spain; ^5^Research and Development Functional Food CentreGranada, Spain

**Keywords:** chickpea, *Cicer arietinum*, hepatoprotection, antioxidation, cytotoxicity, LD_50_

## Abstract

Chickpea (*Cicer arietinum*) is a legume of the family Fabaceae, subfamily Faboideae. In Egypt, chickpea seeds are usually consumed at raw green and tender stage, or in the form of mature dry seeds. In our previous study, ‘Giza 1’ seeds exhibited stronger antioxidant activity and higher total phenol content than those from other Egyptian cultivars. In order to assess the biological potential of ‘Giza 1’ seeds *in vivo*, the extraction procedure was reproduced here. The extract was standardized using liquid chromatography coupled to diode array detector and tandem mass spectrometry (MS/MS) to evaluate their hepatoprotective effect on carbon tetrachloride (CCl_4_)-induced hepatotoxicity in rats and acute toxicity. Administration of the extract to rats in doses up to 2 g/Kg) did not cause any mortalities or observable signs of toxicity. Further, the plant extract showed a strong hepatoprotective activity based on assessing serum alanine aminotransferase, aspartate aminotransferase, and alkaline phosphatase and levels of albumen, globulin, total protein, total cholesterol, high density lipoprotein, triglycerides, and low density lipoprotein. The antioxidative activity was evaluated by assessing hepatic catalase and superoxide dismutase activity as well as reduced glutathione, and malondialdehyde levels. Additionally, anti-inflammatory activity was observed as the extract significantly lowered the hepatic tumor necrosis factor α content. Histopathological examination of liver tissues indicated that the extract-treated animals showed almost normal hepatic architecture with fewer pathological changes. In conclusion, the current results suggest that the chickpea extract possesses an excellent safety profile with very low acute toxicity. Also, it exhibits a significant hepatoprotective effect against CCl_4_-induced liver injury in rats. This can be attributed, at least partly, to the antioxidant and anti-inflammatory activity of the isoflavones and phenolic acids content of the extract.

## Introduction

Liver is one of the most vital organs in the human body which is involved in the regulation of various biochemical functions (Wolf, 1999; Raj and Gothandam, 2014). It bears noting that the lack of proper management of liver disorders by regular medicinal system gives more relevance for the development of effective and safe naturally derived hepatoprotective drugs. A plethora of studies suggest that the consumption of fruits and vegetables rich in natural antioxidants reduce the risk of chronic hepatic diseases (Sabir et al., 2012).

Carbon tetrachloride (CCl_4_) is a potent environmental toxicant inducing severe hepatic damage *via* the generation of highly reactive free radicals. These radicals initiate lipid peroxidation by the covalent binding to phospholipid membranes which harm cellular permeability and finally leading to severe cellular damage (Anusuya et al., 2010; Akther et al., 2014; Raj and Gothandam, 2014). The second damage of the liver occurs due to inflammatory responses which are initiated by Kupffer cells activation releasing proinflammatory mediators such as tumor necrosis factor-alpha (TNF-α). They stimulate other hepatic cells to attract and activate circulating inflammatory cells (Breikaa et al., 2013). In this context, plants rich in natural antioxidants, in particular, phenolic compounds have free radical scavenging ability with enhancement of the endogenous antioxidant enzymes *viz.* superoxide dismutase (SOD), catalase (CAT) as well as non-enzymatic antioxidants as reduced glutathione (GSH) (Akther et al., 2014; Raj and Gothandam, 2014). Therefore, antioxidants rich plants could be potent hepatoprotective agents (Zeashan et al., 2009; Parenti et al., 2015).

Among leguminous foods, chickpea (*Cicer arietinum* L.) is considered a basic food in many countries. They represent a source of carbohydrates, dietary proteins among other nutrients (Jukanti et al., 2012). Several studies discussed secondary metabolites of chickpeas which include several phytochemical classes with a focus on phenolic compounds (Ruiz et al., 1996; Aguiar et al., 2007; Sreerama et al., 2010; Aguilera et al., 2011; Srivastava and Vasishtha, 2012; Wu et al., 2012; Mekky et al., 2015). Our previous study, revealed that ‘Giza 1’ contained the highest total phenol content and the most potent *in vitro* antioxidant activity when compared with other Egyptian cultivars (Mekky et al., 2015).

Therefore, this work was undertaken on a selected chickpea cultivar to assess its *in vivo* hepatoprotective activity. Furthermore, the extract was standardized by liquid chromatography (LC) coupled to diode array detection (DAD) and tandem mass spectrometry (MS/MS).

## Materials and Methods

### Chemicals

Alanine aminotransferase (ALT), aspartate aminotransferase (AST), triglycerides and high density lipoprotein (HDL)-cholesterol kits were purchased from Vitro Scient, Cairo, Egypt. Alkaline phosphatase (ALP) kit was purchased from Greiner Diagnostic GmbH, Bahlingen, Germany. Albumin, total protein and total cholesterol were purchased from Spectrum Diagnostics, Cairo, Egypt. Reduced GSH, superoxide dismutase (SOD), malondialdehyde and catalase (CAT) kits were purchased from Bio-Diagnsotic, Giza, Egypt. Rat TNF-α ELISA Kit was purchased from RayBiotech, Inc., Norcross, GA, USA.

Carbon tetrachloride (CCl_4_), eosin-hematoxylin solution, trichrome stain (Masson), formalin solution (neutral buffered, 10%), methyl alcohol, ethyl alcohol, paraffin beeswax refined, and other standards were purchased from Sigma-Aldrich Co., St Louis, MO, USA.

Hepaticum^®^ (Silymarin, reference drug) suspension (Medical Union Pharmaceuticals, Abou Sultan – Ismailia, Egypt).

### Plant Material

Seeds of the Egyptian chickpea cultivar ‘Giza 1,’ were kindly provided and identified by Dr. Mostafa Abdel Moamen and Agriculture Engineer Ahmed Abd Allah, Field Crops Research Institute, Agricultural Research Center, Giza, Egypt.

### Preparation of Chickpea Extract

Succinctly, 500 g of chickpea cultivar ‘Giza 1’ powder were extracted according to method reported by Mekky et al. (2015), which is based on two steps of solid-liquid extraction with methanol:water (50:50, v/v) and subsequently acetone:water (70:30, v/v). Both extracts were combined and evaporated. The yield was 27.97% from the initial weight.

### Chemical Study

#### Analysis of the Chickpea Extract by LC-DAD-MS/MS

Analyses were made with an Agilent 1200 series rapid resolution (Santa Clara, CA, USA), which contained a binary pump, an autosampler and a DAD. The system was coupled to a 6540 Agilent Ultra-High-Definition (UHD) Accurate-Mass Q-TOF LC/MS (Palo Alto, CA, USA) with an electrospray interface.

A core-shell Halo C18 analytical column (150 mm × 4.6 mm, 2.7 μm particle size) was used for separation of phenolic compounds using acidified water (0.5% acetic acid, v/v) and acetonitrile, as mobile phases A and B, respectively, and a constant flow rate of 0.5 mL/min. The gradient program was as follows: 0 min 99% A and 1% B, 5.50 min 93% A and 7% B, 11 min 86% A and 14% B, 17.50 min 76% A and 24% B, 22.50 min 60% A and 40% B, 27.50 min 0% A and 100% B, 29.5 min 99% A and 1% B. The later conditions were maintained for 5.50 min for column re-equilibration. The injection volume was 8 μL.

The MS parameters were: drying nitrogen gas temperature 325°C with flow of 10 L/min; nebulizer pressure 20 psig; sheath gas temperature 400°C with flow of 12 L/min; capillary voltage 4000 V, nozzle voltage 500 V, fragmentor voltage 130 V, skimmer voltage 45 V, octopole radiofrequency voltage 750 V. Data acquisition at 2.5 Hz was obtained in both the centroid and profile modes using MassHunter Workstation software (Agilent technologies). The spectra were acquired in the negative ionization mode from *m/z* 70 to 1100. The detection window was set to 100 ppm. Reference mass correction was performed with a continuous infusion of Agilent TOF biopolymer analysis mixture: trifluoroacetic acid ammonium salt (*m/z* 112.9856) and hexakis (1H, 1H, 3H-tetrafluoropropoxy) phosphazine (*m/z* 980.0164, acetic adduct).

For further data analysis, MassHunter Qualitative Analysis B.06.00.qa. (Agilent technologies) was employed. The characterization strategy was based on the generation of a candidate formula for molecular ions with a mass accuracy limit of 5 ppm and considering the isotopic pattern. Thus, the MS limit was of ≥80. Afterward, the molecular formula of the candidate, its retention time (RT), UV, and MS/MS spectra were matched with those reported in literature and databases. Consequently, the following chemical structure databases were consulted: PubChem^1^, ChemSpider^2^, SciFinder Scholar^3^, Reaxys^4^, Phenol-Explorer^5^, KNApSAcK Core System^6^, MassBank^7^, and METLIN Metabolite Database^8^.

### Biological Study

#### Animals

The study was conducted according to the National Institute of Health for Care and Use of Laboratory Animals (Publication No. 85-23, revised 1985) and approved by the local Research Ethical Committee at the Faculty of Pharmacy, Cairo University (Egypt). Male wistar albino rats weighing 150–250 g were obtained from the Nile Co. for Pharmaceuticals and Chemical industries, Cairo, Egypt. The animals were kept in Faculty of Pharmacy, Egyptian Russian University animal house in an air-conditioned atmosphere (25 ± 3°C) and kept on a standard diet and water *ad libitum*. Standard diet pellets (El-Nasr Co., Abu-Zaabal, Al-Qalyubiyah, Egypt) contained not less than 20% protein, 5% fiber, 3.5% fat, 6.5% ash, and a vitamins mixture.

#### Acute Toxicity Study

The extract of chickpea cultivar ‘Giza 1’ was administrated orally to four groups of rats (six animals each). It was given in doses ranged from 250 to 2000 mg/kg. The rats were observed for 72 h in accordance with Banda et al. (2013).

#### Hepatoprotective Study

The extract was tested for the possible hepatoprotective effect using a model of CCl_4_ induced hepatotoxicity. Rats were randomized into five groups (eight animals each) and treated for 7 days according to reported literature (Shanmugasundaram and Venkataraman, 2006; Banda et al., 2013; Breikaa et al., 2013; Raj and Gothandam, 2014) as follows. Group 1 (control): rats of this group received distilled water (1 ml/kg p.o.) every day and received olive oil (1 ml/kg, i.p) on second, fourth and sixth days. Group 2 (CCl_4_): rats of this group received distilled water (1 ml/kg p.o.) every day + CCl_4_ [2 ml/kg, 1:1 (v/v) in olive oil i.p.] on second, fourth and sixth days. Groups 3 (silymarin): rats of this group received silymarin (100 mg/kg p.o.) every day + CCl_4_ [2 ml/kg, 1:1 (v/v) with olive oil i.p.] on second, 4th and 6th days. Group 4: rats of this group received extract (CA250: 250 mg/kg p.o.) every day + CCl_4_ [2 ml/kg, 1:1 (v/v) with olive oil i.p.] on second, fourth and sixth days. Group 5: rats of this group received extract (CA500:500 mg/kg p.o.) every day + CCl_4_ [2 ml/kg, 1:1 (v/v) with olive oil i.p.] on second, fourth and sixth days.

Blood was collected from retro plexus after 24 h of last dose. It was allowed to clot and centrifuged at 2500 rpm for separation of serum. All the animals were sacrificed by cervical dislocation immediately after blood collection. Liver tissues were excised for biochemical and histopathological analyses.

#### Estimation of Hepatic Indices

Hepatic index (HI) was calculated from the ratio of liver weight with respect to the total body weight

$$HI=\frac{liver\,\;weight}{body\,\;weight}\times \text{100 (Zhang et al., 2014).}$$

Serum levels of ALT, AST, ALP, albumin, and total protein were determined following instructions provided by the manufacturer (Breikaa et al., 2013). Whereas, the serum level of globulins and the albumin/globulins (A/G) ratio were calculated according to Kingsley (1939) equations;

Serum⁢ globulins⁢ conc.⁢ (g/dL)=Total⁢ protien⁢ conc.−Albu⁢min⁡ conc.

and

$$A/G/\;\;Ratio=\frac{Albu\,\min\;conc.}{Globulins\,\;conc.}$$

#### Estimation of Lipid Profile

Serum levels of total cholesterol, triglycerides (TG) and HDL-cholesterol were determined following instructions provided by the manufacturer (Breikaa et al., 2013). Whereas, the serum level of low density lipoprotein cholesterol (LDL) was determined according to Freidewald formula (Friedewald et al., 1972), where;

Serum⁢ LDL⁢ Conc.⁢ (mg/dL)=Total⁢ cholesterol−HDL−(TG/5)

#### Assessment of Oxidative Stress Markers

The hepatic tissue was homogenated in 10 mL of cold potassium phosphate buffer (100 mM, pH 7, containing 2 mM EDTA per gram tissue). The homogenate was centrifuged at 4000 rpm and 4°C for 15 min. The supernatants were stored at -80°C for analysis. Hepatic tissue levels of GSH reduced content (Beutler et al., 1963), super oxide dismutase activity (Nishikimi et al., 1972), catalase activity (Aebi, 1984) and of thiobarbituric acid reactive substances indicative to malondialdehyde content (Kei, 1978) were determined following instructions provided by the manufacturer.

#### Assessment of Tumor Necrosis Factor-Alpha (TNFα)

Determination of TNFα was performed according to Bonavida (1991) and was carried out according to instructions provided by the manufacturer.

#### Histopathological Examination using Hematoxylin and Eosin (H and E) and Masson’s Trichrome

Liver samples from treated animals were processed for light microscopy. The liver specimens were stained according to the method described by Bancroft and Gamble (2008). Liver specimens were taken from the right lobe and fixed in 10% formalin for 24 h then washing was done with tap water. Serial dilutions of alcohol (methyl, ethyl, and absolute ethyl) were used for dehydration. Specimens were cleared in xylene embedded in paraffin at 56°C in hot air oven for 24 h. Paraffin bees wax tissue blocks were prepared for sectioning at four microns thickness by sledge microtome. The obtained tissue sections were collected on glass slides and, deparaffinized. After that, sections were stained with Hematoxylin and Eosin (H and E) for routine histological examination and Masson’s trichrome for demonstration of collagen fibers.

### Statistical Analysis

Microsoft Excel 2007 (Redmond, WA, USA) was employed for statistical analysis of the data with the level of significance set at 95%. One-way analysis of variance (ANOVA) was performed to assess statistical differences between extractions using Tukey *post hoc* test and the software IBM SPSS Statistics 22 (Armonk, NY, USA).

## Results and Discussion

### Phenolic Profiling by RP-HPLC-DAD-ESI-QTOF-MS and -MS/MS

**FIGURE 1:**
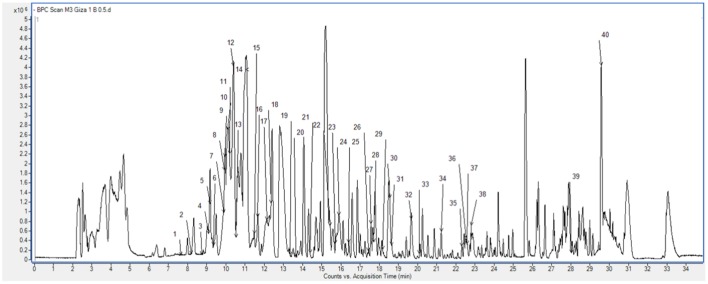
**Base peak chromatogram of the Egyptian cultivar ‘Giza 1’ showing the characterized phenolic compounds peaks numbers**.

The analysis of the extract of ‘Giza 1’ cultivar revealed the presence of phenolic acids and flavonoids (Supplementary Table S1). The compounds were characterized by comparison of RT, molecular formula and UV spectra and MS/MS fragmentation patterns with those of literature, as denoted before (Mekky et al., 2015). Remarkably, the main phenolic compounds were the isomers of dihydroxybenzoic acid hexoside at RT 10.09 and 10.39 min, with relative amounts of 15.803 and 16.883%, the hydroxybenzoic acid hexoside pentoside at RT 11.01 min and relative amount of 12.566%, and followed by the isoflavone biochanin A (RT 29.66 min, 9.454%) (Supplementary Table S1; **Figure 1**).

In addition, hydroxycinnamic acids were represented by hexosides of ferulic acid and sinapic acid, and other characteristic flavonoids included derivatives of kaempferol (flavonol), aromadendrin (flavanonol), and naringenin (flavanone), among others.

### Acute Toxicity Study

No mortality was detected in all the groups received the chickpea extract up to 2000 mg/Kg observed for 72 h. This complies with the previous results of Indian chickpea cultivars (Banda et al., 2013), which indicated the safety of the seeds extract.

### Estimation of Hepatoprotection Activity

The intraperitoneal administration of CCl_4_ provoked significantly liver damage which was observed clearly by the elevated levels of the hepatic index as well as ALT, AST, and ALP compared to the control group (**Table 1**). The elevation of hepatic index is a marker of liver hypertrophy (increase in liver weight with respect to the total body weight) (Yachi et al., 2010). In the same manner, the raised levels of liver enzymes in the serum indicated their release from damaged hepatic cells associated with hepatic injury (Yachi et al., 2010; Banda et al., 2013; Breikaa et al., 2013; Akther et al., 2014). The oral administration of chickpea ‘Giza 1’ extract exerted significant hepatoprotective activity by reduction of the aforementioned parameters compared to the reference drug silymarin (**Table 1**), this complies with results described previously (Banda et al., 2013; Santhoshi et al., 2013; Sri Ramachandra et al., 2014). This activity may be attributed to the characterized phenolics in the extract, *viz* hydroxybenzoic acid derivatives and biochanin A (Kinjo et al., 2006).

**Table 1:** Hepatoprotective effect of reference drug silymarin, chickpea extract (CA250 and CA500) against carbon tetrachloride (CCl_4_) induced hepatotoxicity on hepatic index and serum levels of aminotransferase (ALT), aminotransferase (AST), and alkaline phosphatase (ALP), albumin, total protein, and globulins, and A/G ratio.

Group	HI	ALT (U/L)	AST (U/L)	ALP (U/L)	Albumin (g/dL)	Total Protein (g/dL)	Globulins (g/dL)	A/G
**Control**	4.130 ± 0.172^b^	23.679 ± 2.710^b^	19.548 ± 2.542^b^	132.132 ± 26.231^b^	3.702 ± 0.148^b^	5.230 ± 1.052	1.240 ± 0.334^b^	2.987
**CCl_4_**	4.848 ± 0.266^a^	91.025 ± 18.760^a^	49.482 ± 6.210^a^	239.246 ± 36.033^a^	2.918 ± 0.048^a^	6.083 ± 0.968	3.405 ± 8.858^a^	0.857
**Silymarin + CCl_4_**	4.378 ± 0.335^ab^	32.535 ± 7.912^b^	24.093 ± 0.465^b^	152.685 ± 19.678^b^	3.271 ± 0.071^ab^	5.176 ± 0.934	1.524 ± 8.339^b^	2.147
**CA250 + CCl_4_**	4.563 ± 0.225^ab^	36.283 ± 1.586^b^	27.911 ± 5.994^ab^	161.376 ± 22.787^b^	3.047 ± 0.276^ab^	5.825 ± 0.600	2.778 ± 3.600^a^	1.097
**CA500 + CCl_4_**	4.208 ± 0.405^b^	31.465 ± 7.081^b^	22.352 ± 5.389^b^	153.933 ± 29.661^b^	3.538 ± 0.071^b^	4.818 ± 0.448	1.422 ± 8.314^b^	2.488

*Data are the mean ± standard deviation (n = 8); a, b, or c significantly different at p < 0.05 using ANOVA followed by Tukey as a post hoc test.*

Similarly, the intraperitoneal injection of CCl_4_ significantly decreased the level of serum albumin by 21.6%, indicating alteration of albumin synthesis in liver associated with hepatic intoxication and impairment of liver functions (Zafar and Ali, 1998). This was accompanied with the elevated levels of globulins by 77% and consequently the reduction of albumin/globulins (A/G) ratio compared to the control group (Thirunavukkarasu and Sakthisekaran, 2003; Akther et al., 2014) (**Table 1**). Remarkably, oral administration of the higher dose of chickpea ‘Giza 1’ extract (CA500), rich in phenolics, significantly restored the levels of serum albumin, and globulins to the control group indicating hepatoprotective activity with restoration of synthesis of albumin in liver (**Table 1**).

### Assessment of Lipid Profile

The induction of hepatotoxicity with CCl_4_ significantly increased the level of serum total cholesterol by 125%, LDL and triglycerides with the significant decrease of serum level of HDL which was attributed to the alteration of lipoprotein metabolism in the liver (Ravikumar et al., 2010; Yachi et al., 2010; Breikaa et al., 2013; Akther et al., 2014) (**Table 2**). Remarkably, the oral administration of the higher dose of chickpea ‘Giza 1’ extract (CA500) significantly restored the levels of serum total cholesterol, LDL, HDL, and triglycerides to the standard group indicating hepatoprotective activity with preservation of cellular integrity and antilipidemic effect. These results complying with previous reports on chickpeas sprout (Sharma, 1987; Harini et al., 2015) (**Table 2**).

**Table 2:** Hepatoprotective effect of reference drug silymarin, chickpea extract (CA250 and CA500) against CCl_4_ induced hepatotoxicity on serum levels of total cholesterol, triglycerides, high density lipoprotein (HDL) and low density lipoprotein (LDL).

Group	Total Cholesterol (mg/dl)	Triglycerides (mg/dL)	HDL (mg/dL)	LDL (mg/dL)
**Control**	19.886 ± 83.222^b^	4.327 ± 84.130^b^	12.583 ± 56.602^b^	1.708 ± 9.822^b^
**CCl_4_**	37.747 ± 187.500^a^	21.480 ± 185.792^a^	2.896 ± 18.123^a^	11.524 ± 107.319^a^
**Silymarin + CCl_4_**	27.052 ± 110.143^b^	7.216 ± 118.378^c^	7.995 ± 31.320^c^	3.500 ± 31.254^c^
**CA250 + CCl_4_**	27.700 ± 115.545^b^	1.586 ± 141.389^c^	5.917 ± 25.647^c^	11.768 ± 43.742^c^
**CA500 + CCl_4_**	25.822 ± 102.600^b^	3.327 ± 130.556^c^	7.044 ± 29.207^c^	7.421 ± 33.348^c^

*Data are the mean ± standard deviation (n=8); a, b, or c significantly different at p < 0.05 using ANOVA followed by Tukey as a post hoc test*.

### Assessment of *In vivo* Antioxidant Activity

**FIGURE 2:**
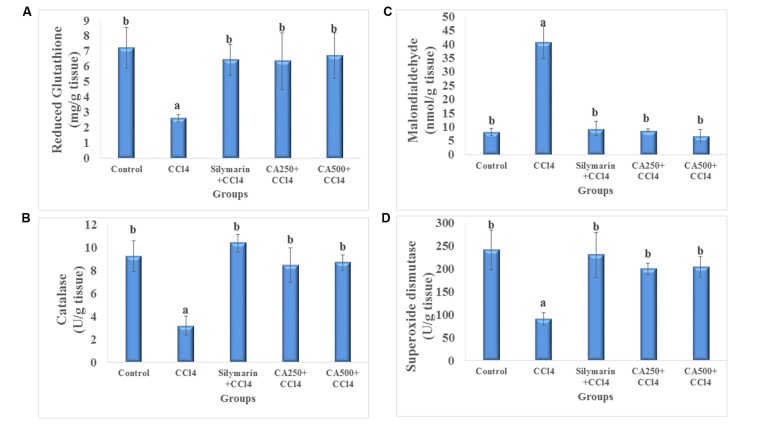
***In vivo* antioxidant activity of reference drug silymarin, CA250 and CA500 against CCl_4_ induced hepatotoxicity on liver tissue levels of (A) reduced glutathione, (B) malondialdehyde, (C) catalase, and (D) superoxide dismutase.** Data are the mean ± standard deviation (*n* = 8); *a* or *b* significantly different at *p* < 0.05 using ANOVA followed by Tukey as a *post hoc* test.

The *in vivo* antioxidant activity was determined *via* the assay of liver endogenous antioxidants *viz.* non-enzymatic (GSH) and enzymatic (CAT and SOD), and MDA as a marker of lipid peroxidation. The administration of CCl_4_ significantly decreased the levels of endogenous antioxidants GSH by 63.6%, CAT by 65.5%, and SOD by 62.2% with the significant increase of hepatic level of MDA by 392.8% or four folds (Breikaa et al., 2013; Akther et al., 2014) (**Figure 2**). The oral administration of chickpea ‘Giza 1’ extract (CA250 and CA500) significantly restored the levels of them nearly to values of the control group indicating *in vivo* antioxidant activity (**Figure 2**). This complies with the data reported by Sri Ramachandra et al. (2014) on aerial parts of chickpea extract (Sri Ramachandra et al., 2014). Consequently, the antioxidant and hepatoprotective activity could be associated with the phenolics present in ‘Giza 1’ cultivar according to Kinjo et al. (2006).

### Assessment of Inflammatory Marker (TNFα)

**FIGURE 3:**
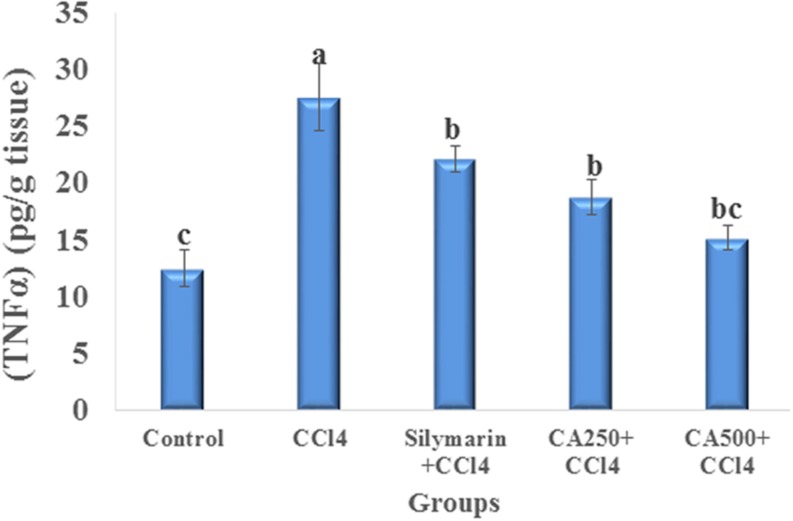
**Anti-inflammatory effect of reference drug silymarin, CA250 and CA500 against CCl_4_ induced hepatotoxicity on tumor necrosis factor alpha (TNFα).** Data are the mean ± standard deviation (*n* = 8); *a*, *b*, or *c* significantly different at *p* < 0.05 using ANOVA followed by Tukey as a *post hoc* test.

The induction of hepatic injury with CCl_4_ stimulates Kupffer cells, which are fixed macrophages, to produce, and secrete proinflammatory mediators as TNFα. In turn, TNFα stimulates the release of cytokines from the macrophages and induces phagocyte oxidative metabolism causing secondary liver damage. This inflammation spreads by stimulation of the endothelium, and leads to localized migration of monocytes and neutrophils (Yachi et al., 2010; Breikaa et al., 2013). In this study, CCl_4_ intoxication increased significantly the level of TNFα by 121.3% (Yachi et al., 2010; Breikaa et al., 2013) (**Figure 3**). In fact, the oral administration of the higher dose of chickpea ‘Giza 1’ extract (CA500) significantly restored the level of TNFα (15.150 ± 1.061 pg/g tissue) to the direction of the control group more effectively than the standard silymarin indicating hepatoprotective and anti-inflammatory activity complying with the previous study on Indian chickpea cultivar (Sandeep et al., 2012) (**Figure 3**).

### Histopathological Examination

**FIGURE 4:**
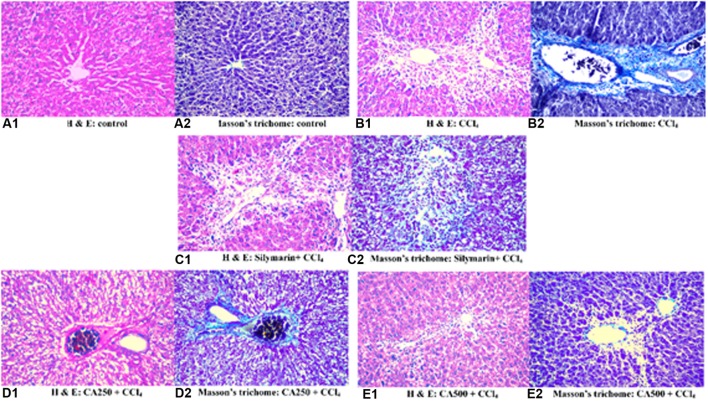
**Histopathological sections of liver with CCl_4_ induced hepatotoxicity stained with (1) hematoxylin and eosin and (2) Masson’s trichome; (A) control group, (B) CCl_4_ group, (C) silymarin + CCl_4_ group, (D) CA250 + CCl_4_ group, (E) CA500 + CCl_4_ group**.

The different groups of rats were studied for cellular architecture. The control group showed normal histological structure of the central vein and surrounding hepatocytes in the parenchyma (**Figures 4A1,A2**). Group 2 (CCl_4_) showed centrilobular necrosis in the hepatocytes surrounding the central veins in diffuse manner all over the hepatic parenchyma associated with vacuolar degeneration and fatty change in the adjacent (**Figure 4B1**). Upon staining with Masson’s trichome, the blue color of collagen fibers was visualized indicating fibrosis surrounding the central portal vein (**Figure 4B2**). Regarding group 3 (silymarin + CCl_4_), the portal area showed congestion in the portal vein while the hepatic parenchyma had centrilobular necrosis in the hepatocytes surrounding the central vein to less extent (**Figures 4C1,C2**).

In the same manner, group 4 treated with the lower dose of the extract (CA250 + CCl_4_) showed degeneration of hepatocytes in diffuse manner all over the hepatic parenchyma associated with congestion in the portal vein and dilatation in the bile duct (**Figures 4D1,D2**). Upon treatment with the higher dose of the extract in group 5 (CA500 + CCl_4_), a more normal architecture of liver with fewer hepatocytes showing fatty change. A fewer number of hepatocytes surrounding the central vein had necrobiosis (**Figures 4E1,E2**). The histopathological examination is complying with the previous reports on chickpeas (Banda et al., 2013; Santhoshi et al., 2013; Sri Ramachandra et al., 2014).

Although the latter studies have shown the hepatoprotective role of chickpeas extracts, their individual phenolic constituents have never been characterized. Alternatively, our results revealed the presence of biochanin A in ‘Giza 1’ chickpea extract. This isoflavone exerts hepatoprotective activity (Belguith-Hadriche et al., 2016) thanks to its antioxidant, anti-inflammatory, and immunomodulatory actions. Isoflavones such as daidzin, genistin, daidzein, and genistein from soy (Sarhan et al., 2012) also prevented CCl_4_-induced hepatotoxicity in rats. Concerning hydroxybenzoic derivatives, previous studies suggest that this type of compounds, in their aglycone form, may suppress hepatic fibrosis in chronic liver injury (Sarhan et al., 2012). Moreover, Belguith-Hadriche et al. (2016) have shown that a fig extract rich in dihydroxybenzoic acid glycosides and rutin had a significant hypocholesterolemic effect by decreasing serum total cholesterol, TG, LDL, and increasing HDL cholesterol. Therefore, the presence of these phenolic compounds in ‘Giza 1’ chickpea extract could contribute to the anti-inflammatory action, hepatoprotective activity and restoration of liver architecture *in vivo.*

## Conclusion

Our results revealed that no treatment-related toxicity was detected after the administration of ‘Giza 1’ chickpea extract. This extract exhibited a strong hepatoprotective activity *in vivo* based on measurement of TNF-α, levels of albumen, globulin, total protein and lipid profile, and oxidative status. The hepatoprotective activity was further confirmed from the histopathological examination. Therefore, further bio-guided studies are required to evaluate the individual contribution of chickpea isoflavones and hydroxybenzoic derivatives in hepatoprotective activity.

## Author Contributions

All authors listed, have made substantial, direct and intellectual contribution to the work, and approved it for publication.

## Conflict of Interest Statement

The authors declare that the research was conducted in the absence of any commercial or financial relationships that could be construed as a potential conflict of interest. The reviewer CP and handling Editor declared their shared affiliation, and the handling Editor states that the process nevertheless met the standards of a fair and objective review.

## Footnotes

1 http://pubchem.ncbi.nlm.nih.gov

2 http://www.chemspider.com

3 https://scifinder.cas.org

4 http://www.reaxys.com

5 http://www.phenol-explorer.eu/

6 http://kanaya.naist.jp/knapsack_jsp/top.html

7 http://www.massbank.jp

8 http://metlin.scripps.edu
