# Supplementary material for: Hepatoprotective Effect and Chemical Assessment of a Selected Egyptian Chickpea Cultivar
Source: Front Pharmacol. 2016 Sep 28;7:344. doi: 10.3389/fphar.2016.00344 (PMC5040119; doi:10.3389/fphar.2016.00344)
Supplement: Supplementary file 1 [file Table_1.DOCX]

Table S1. Phenolic compounds characterized in Egyptian chickpea extract of ‘Giza 1’ cultivar.

| Peak No | RT (min) | Experimental *m/z*^a^ [M-H]^-^ | Theoretical mass (M) | Molecular formula (M) | Error (ppm) | Error (mDa) | Score | Main fragments | UV (nm) | Compound | Subclass | % of relative amount |
| --- | --- | --- | --- | --- | --- | --- | --- | --- | --- | --- | --- | --- |
| 1 | 7.65 | 331.0674 | 332.0743 | C_13_H_16_O_10_ | -0.1 | 0.0 | 90.8 | 313.0562, 169.0136, 168.0065, 125.0233 | 230, 256 | Gallic acid hexoside I | Hydroxybenzoic acid | 0.398 |
| 3 | 8.20 | 329.0886 | 330.0951 | C_14_H_18_O_9_ | -2.0 | -0.7 | 82.2 | 167.0316, 122.0367 | 258 | Vanillic acid hexoside | Hydroxybenzoic acid | 0.175 |
| 4 | 8.76 | 331.0673 | 332.0743 | C_13_H_16_O_10_ | -0.4 | -0.1 | 99.2 | 313.0569, 169.0138, 168.0058, 125.0242 | 254 | Gallic acid hexoside II | Hydroxybenzoic acid | 1.231 |
| 5 | 9.09 | 299.0777 | 300.0845 | C_13_H_16_O_8_ | -1.4 | -0.4 | 98.8 | 137.0245, 93.0346 | N.D. | Hydroxybenzoic acid hexoside I | Hydroxybenzoic acid | 1.317 |
| 6 | 9.20 | 299.0777 | 300.0845 | C_13_H_16_O_8_ | -1.4 | -0.4 | 98.5 | 137.0245, 93.0347 | 248 | Hydroxybenzoic acid hexoside II | Hydroxybenzoic acid | 1.697 |
| 7 | 9.34 | 461.1296 | 462.1373 | C_19_H_26_O_13_ | 1.7 | 0.8 | 93.4 | 417.1397, 285.0992, 123.0459 | N.D. | Vanillic acid hexoside pentoside I | Hydroxybenzoic acid | 0.137 |
| 8 | 9.90 | 315.0733 | 316.0794 | C_13_H_16_O_9_ | -3.5 | -1.1 | 95.5 | 153.0195, 152.0117, 109.0299, 108.0217 | 254, 314 | Dihydroxybenzoic acid hexoside I | Hydroxybenzoic acid | 3.818 |
| 9 | 9.93 | 331.0670 | 332.0743 | C_13_H_16_O_10_ | 0.3 | 0.1 | 99.6 | 313.0576, 169.0148, 168.0074, 125.0250 | N.D. | Gallic acid hexoside III | Hydroxybenzoic acid | 3.555 |
| 10 | 9.94 | 431.1205 | 432.1286 | C_18_H_24_O_12_ | -2.1 | -0.9 | 97.4 | 137.0244, 93.0349 | N.D. | Hydroxybenzoic acid hexoside pentoside I | Hydroxybenzoic acid | 1.486 |
| 11 | 10.09 | 315.0721 | 316.0794 | C_13_H_16_O_9_ | 0.4 | 0.1 | 99.2 | 153.0183, 152.0119, 109.0119, 108.0219 | 236, 314 | Dihydroxybenzoic acid hexoside II | Hydroxybenzoic acid | 15.803 |
| 12 | 10.19 | 461.1298 | 462.1373 | C_19_H_26_O_13_ | 1.1 | 0.5 | 97.4 | 315.0782, 153.0227 | N.D. | Dihydroxybenzoic acid hexoside deoxyhexoside | Hydroxybenzoic acid | 1.438 |
| 13 | 10.39 | 315.0726 | 316.0794 | C_13_H_16_O_9_ | -1.2 | 0.4 | 98.3 | 153.0195, 152.0116, 109.0297, 108.0220 | 240, 314 | Dihydroxybenzoic acid hexoside III | Hydroxybenzoic acid | 16.883 |
| 14 | 10.53 | 431.1204 | 432.1286 | C_18_H_24_O_12_ | -1.8 | -0.8 | 97.9 | 299.0795, 137.0250, 93.0353 | 252 | Hydroxybenzoic acid hexoside pentoside II | Hydroxybenzoic acid | 0.642 |
| 15 | 11.01 | 431.1204 | 432.1286 | C_18_H_24_O_12_ | -2.0 | -0.9 | 97.1 | 299.0892, 137.0311, 93.0399 | 250 | Hydroxybenzoic acid hexoside pentoside III | Hydroxybenzoic acid | 12.566 |
| 16 | 11.49 | 461.1295 | 462.1373 | C_19_H_26_O_13_ | 1.3 | 0.6 | 99.1 | 329.0879, 167.0347, 152.0111 | 254, 292 | Vanillic acid hexoside pentoside II | Hydroxybenzoic acid | 2.112 |
| 17 | 11.65 | 315.0728 | 316.0794 | C_13_H_16_O_9_ | -2.0 | -0.6 | 97.5 | 153.0200, 109.0299 | 238, 307 | Dihydroxybenzoic acid hexoside IV | Hydroxybenzoic acid | 2.736 |
| 18 | 12.13 | 447.1147 | 448.1217 | C_18_H_24_O_13_ | -0.5 | -0.2 | 99.6 | 315.0729, 153.0195, 152.0119, 109.0296, 108.0221 | 257, 305 | Dihydroxybenzoic acid hexoside pentoside I | Hydroxybenzoic acid | 6.693 |
| 19 | 12.35 | 447.1143 | 448.1217 | C_18_H_24_O_13_ | 0.4 | 0.2 | 99.1 | 315.0723, 153.0186, 152.0113, 109.0289, 108.0215 | 231, 316 | Dihydroxybenzoic acid hexoside pentoside II | Hydroxybenzoic acid | 7.480 |
| 22 | 13.34 | 285.0616 | 286.0689 | C_12_H_14_O_8_ | 0.0 | 0.0 | 98.8 | 153.0182, 152.0114, 109.0927, 108.0212 | 268 | Dihydroxybenzoic acid pentoside | Hydroxybenzoic acid | 0.268 |
| 23 | 13.64 | 609.1466 | 610.1534 | C_27_H_30_O_16_ | -0.6 | -0.4 | 97.8 | 447.0943, 285.0412, 284.0325, 151.0031 | 243, 314, 342 | Kaempferol dihexoside I | flavonol | 0.163 |
| 24 | 14.39 | 355.1043 | 356.1107 | C_16_H_20_O_9_ | -2.0 | -0.7 | 82.3 | 193.0509, 149.0607 | 232, 291, 314 | Ferulic acid hexoside | Hydroxycinnamic acid | 0.298 |
| 28 | 15.42 | 153.0191 | 154.0266 | C_7_H_6_O_4_ | 1.2 | 0.2 | 99.5 | 109.0297 | 248, 322 | Dihydroxybenzoic acid I | Hydroxybenzoic acid | 0.388 |
| 29 | 15.72 | 137.0247 | 138.0317 | C_7_H_6_O_3_ | -1.6 | -0.2 | 99.4 |  | 256 | *p*-hydroxybenzoic acid* | Hydroxybenzoic acid | 0.265 |
| 31 | 15.90 | 401.0740 | 402.0798 | C_16_H_18_O_12_ | -0.3 | -0.1 | 97.3 | 357.0827, 315.0730, 153.0204, 152.0125, 109.0309, 108.0230 | 230, 280 | Dihydroxybenzoic acid malonyl hexoside | Hydroxybenzoic acid | 1.118 |
| 34 | 16.40 | 827.1894 | 828.1960 | C_35_H_40_O_23_ | -0.4 | 0.0 | 99.0 | 783.2006, 621.1472, 447.0898, 285.0389, 284.0333, 151.0035 | 265, 353 | Kaempferol malonyl dihexoside pentoside I | flavonol | 0.662 |
| 39 | 17.43 | 385.1138 | 386.1213 | C_17_H_22_O_10_ | 0.7 | 0.3 | 98.4 | 223.0616, 208.0375, 191.0198, 179.0139 | 256 | Sinapic acid hexoside | Hydroxycinnamic acid | 0.105 |
| 42 | 17.57 | 827.1887 | 828.1960 | C_35_H_40_O_23_ | 0.4 | 0.3 | 99.0 | 783.2003, 621.1499, 447.0977, 285.0419, 284.0337, 151.0025 | 348 | Kaempferol malonyl dihexoside pentoside II | flavonol | 0.188 |
| 43 | 17.71 | 695.1478 | 696.1538 | C_30_H_32_O_19_ | -1.7 | -1.2 | 97.7 | 651.1556, 489.1038, 447.0923, 446.0851, 285.0409, 151.0023, 131.0714 | 266, 349 | Kaempferol 3-*O*-β-D-(6''-malonyl-) glucopyranoside-7-*O*-β-D-glucopyranoside I | flavonol | 0.158 |
| 46 | 18.17 | 727.2097 | 728.2164 | C_32_H_40_O_19_ | -0.8 | -0.6 | 99.2 | 565.1451, 445.1034, 433.1029, 271.0577, 151.0039, 145.0297 | N.D. | Naringenin dihexoside pentoside | Flavanone | 0.751 |
| 48 | 18.54 | 449.1089 | 450.1162 | C_21_H_22_O_11_ | 0.2 | 0.1 | 97.0 | 287.0563, 269.0450, 259.0609, 153.0183, 151.0030 | N.D. | Aromadendrin hexoside | Flavanonol | 0.144 |
| 49 | 18.64 | 609.1466 | 610.1534 | C_27_H_30_O_16_ | -0.5 | -0.3 | 99.3 | 447.0942, 446.0869, 285.0416, 283.0260, 255.0309, 151.0047 | 264, 346 | Kaempferol dihexoside II | flavonol | 0.586 |
| 54 | 19.69 | 695.1475 | 696.1538 | C_30_H_32_O_19_ | -1.5 | -1.0 | 95.3 | 651.1729, 489.1049, 447.0937, 285.0411, 221.0250, 151.0031 | 266, 343 | Kaempferol 3-*O*-β-D-(6''-malonyl-)glucopyranoside-7-*O*-β-D-glucopyranoside II | flavonol | 0.135 |
| 55 | 20.09 | 725.1935 | 726.2007 | C_32_H_38_O_19_ | -0.1 | -0.1 | 98.8 | 593.1526, 431.1001, 285.0415, 284.0337, 178.9994, 151.0042 | 262, 347 | Kaempferol 3-*O*-lathyroside-7-*O-*α-L-rhamnopyranoside | flavonol | 0.512 |
| 65 | 21.24 | 579.1352 | 580.1438 | C_26_H_28_O_15_ | 0.6 | 0.4 | 99.4 | 447.0949, 285.0402, 284.0329, 255.0301, 151.0193 | 264, 348 | Kaempferol 3-*O*-β-D-apiofuranosyl-(1→2)-β-D-glucopyranoside | flavonol | 1.000 |
| 72 | 22.31 | 665.1351 | 666.1432 | C_29_H_30_O_18_ | 1.6 | 1.0 | 98.1 | 621.1460, 489.1050, 327.0509, 285.0416, 284.0342, 255.0298, 151.0059 | 266, 348 | Kaempferol-3-*O*-[6''-malonyl-β-D-apiofuranosyl-(1→2)-β-D-glucopyranoside] | flavonol | 0.498 |
| 73 | 22.52 | 121.0297 | 122.0368 | C_7_H_6_O_2_ | -1.7 | -0.2 | 99.6 | 77.0394 | 232, 284 | Benzoic acid | Hydroxybenzoic acid | 1.581 |
| 75 | 22.59 | 447.0935 | 448.1006 | C_21_H_20_O_11_ | -0.3 | -0.2 | 99.8 | 327.0521, 285.0406, 284.0331, 255.0299, 227.0352, 151.0033 | 264, 348 | Kaempferol 3-*O*-β-D-glucopyarnoside* | flavonol | 0.610 |
| 77 | 22.80 | 431.0984 | 432.1056 | C_21_H_20_O_10_ | 0.0 | 0.0 | 98.8 | 269.045, 268.0378, 239.0345, 224.0475, 135.0215, 132.0215 | 257, 327 | Genistin [Genistein-7-*​O*-​β-​D-​glucopyranoside] | Isoflavonoid | 0.769 |
| 93 | 28.02 | 299.0567 | 300.0634 | C_16_H_12_O_6_ | -2.1 | -0.6 | 98.5 | 284.0328, 255.0294, 211.0394, 151.0038, 135.0095 | 264, 296 | Pratensein | Isoflavonoid | 0.178 |
| 96 | 29.66 | 283.0616 | 284.0685 | C_16_H_12_O_5_ | -1.3 | -0.4 | 98.5 | 268.0385, 250.0246, 239.0349, 151.0028, 132.0217, 107.0131 | 260, 329 | Biochanin A | Isoflavonoid | 9.454 |

*Identification confirmed by comparison with standards.

Adapted from [Mekky et al. 2015..]
